# Transcription factor networks in aged naïve CD4 T cells bias lineage differentiation

**DOI:** 10.1111/acel.12957

**Published:** 2019-07-01

**Authors:** Bin Hu, Guangjin Li, Zhongde Ye, Claire E. Gustafson, Lu Tian, Cornelia M. Weyand, Jörg J. Goronzy

**Affiliations:** ^1^ Department of Medicine, Division of Immunology and Rheumatology Stanford University Stanford California USA; ^2^ Department of Medicine Palo Alto Veterans Administration Healthcare System Palo Alto California USA; ^3^ Department of Biomedical Data Science Stanford University School of Medicine Stanford California USA

**Keywords:** aging, immunosenescence, interleukin 9, multipotency, T‐cell lineage differentiation, transforming growth factor β

## Abstract

With reduced thymic activity, the population of naïve T cells in humans is maintained by homeostatic proliferation throughout adult life. In young adults, naïve CD4 T cells have enormous proliferative potential and plasticity to differentiate into different lineages. Here, we explored whether naïve CD4 T‐cell aging is associated with a partial loss of this unbiased multipotency. We find that naïve CD4 T cells from older individuals have developed a propensity to develop into TH9 cells. Two major mechanisms contribute to this predisposition. First, responsiveness to transforming growth factor β (TGFβ) stimulation is enhanced with age due to an upregulation of the TGFβR3 receptor that results in increased expression of the transcription factor PU.1. Secondly, aged naïve CD4 T cells display altered transcription factor profiles in response to T‐cell receptor stimulation, including enhanced expression of BATF and IRF4 and reduced expression of ID3 and BCL6. These transcription factors are involved in TH9 differentiation as well as IL9 transcription suggesting that the aging‐associated changes in the transcription factor profile favor TH9 commitment.

## 1 INTRODUCTION

Naïve CD4 T cells distinguish themselves by their enormous proliferative capacity and plasticity. Upon activation, they differentiate into several lineages that provide an array of effector functions and maintain their functional commitment as long‐lived memory cells. Most of the T‐cell functional lineages are characterized by the production of unique sets of cytokines that are tailored to the nature of the inciting assault and that orchestrate specific adaptive immune responses (Zhu, 2017). Multiple lineages have been defined, including TH1, TH2, TH9, TH17, and T follicular helper cells. In addition to these functional subsets characterized by their signature cytokines, naïve T cells can also differentiate into Treg cells that restrain immune responses (Josefowicz, Lu, & Rudensky, 2012). Determinants in lineage decisions include the T‐cell receptor (TCR) signaling strength, the nature of costimulatory signals, and the signals from polarizing cytokines, which activate and upregulate lineage‐defining transcription factors (TF) (Kanno, Vahedi, Hirahara, Singleton, & O'Shea, 2012; Tubo & Jenkins, 2014). T‐cell fates are then frequently stabilized in a forward loop by lineage‐specific cytokines produced by the T cells themselves.

During the first two decades of life, new naïve T cells are produced by the thymus, whereas T‐cell generation during adulthood relies mostly on homeostatic proliferation (den Braber et al., 2012; Goronzy & Weyand, 2017). Even in young adults, <20% of T‐cell generation is from thymic production, which further dwindles to <1% in middle‐aged individuals (Bains, Antia, Callard, & Yates, 2009). Consequently, the number of naïve CD4 T cells and their TCR diversity declines with age. However, homeostatic proliferation is still efficient to maintain a large naïve CD4 compartment with high TCR diversity in healthy older adults (Goronzy & Weyand, 2017; Qi et al., 2014).

What is currently unclear is whether older naïve T cells can maintain their multipotency. Aging is associated with T‐cell‐intrinsic and extrinsic changes that have the potential to influence lineage differentiation. In the murine system, T‐cell aging is associated with cytokine‐driven transition of naïve T cells into virtual memory cells (Renkema, Li, Wu, Smithey, & Nikolich‐Zugich, 2014). Epigenetic studies of human CD8 T cells have provided evidence for loss of stemness as indicated by increased accessibility for bZIP transcription factors with age, a signature that is also a hallmark of differentiation (Moskowitz et al., 2017; Ucar et al., 2017). A similar but less pronounced trend is seen for naïve CD4 T cells (Goronzy, Hu, Kim, Jadhav, & Weyand, 2018). T‐cell phenotypic changes indicating an altered composition with age include the loss of CD31, CR1, and CR2 and the gain of CD25 in subsets of naïve CD4 T cells (den Braber et al., 2012; Kohler & Thiel, 2009; Pekalski et al., 2017). Moreover, TCR activation thresholds and signaling pathways that control differentiation processes change in an age‐dependent pattern. We have shown that the aging‐related decline in miRNA‐181a expression results in overexpression of DUSP6 and SIRT1 and attenuates TCR signaling, favoring TH2 development (Li et al., 2012). Bektas et al. have provided evidence that NF‐κB signaling deteriorates in old CD4 T cells (Bektas et al., 2014). In addition to these T‐cell‐intrinsic changes, the aging process alters the cytokine milieu within the environment, frequently coined as inflamm‐aging (Franceschi et al., 2000). Changes in the cytokine environment not only increase tonic signaling but also induce negative feedback loops that attenuate cytokine‐induced signaling (Goronzy, Li, Yu, & Weyand, 2012; Shen‐Orr et al., 2016).

So far, most immune aging studies have focused on identifying a biased representation of functional T‐cell subsets in the memory population. TH2, TFH, and effector memory T cells have been reported as being enriched in peripheral blood from older individuals; however, these results were inconsistent and difficult to interpret given the multitude of variables in determining the frequencies of functional T‐cell subsets (Gardner & Murasko, 2018). Only a few studies have addressed the question whether age‐related T‐cell‐intrinsic factors in naïve T cells influence TF networks and lineage proclivity. Lee et al. have described increased induction of IL17 production in naïve CD4 T cells, but decreased IL17 production of memory cells in older individuals (Lee et al., 2011). Ramming, Druzd, Leipe, Schulze‐Koops, and Skapenko (2012) have compared histone modifications in promoter regions of T‐bet, GATA3, PU.1, IRF4, and RORC in neonatal and adult naïve T cells and have identified age‐ and differentiation‐related histone modifications in the PU.1 promoter that influenced TH9 differentiation. No difference was seen for the other transcription factors. While the studies by Ramming examined TFs involved in T‐cell differentiation in early age, they raised the possibility that TH9 differentiation may be of interest for immune aging in general. The signature cytokine for TH9 cells is IL‐9, a pleiotropic cytokine that has been initially defined as a growth factor for T cells and mast cells. More recent studies showed IL9 activity on a number of distinct cell types, including airway and intestinal epithelial cells and airway smooth muscle cells, highlighting its role in mucosal inflammation (Kaplan, Hufford, & Olson, 2015).

Here, we describe that naïve CD4 T cells from individuals older than 60 years are prone to differentiate into TH9 cells. Age‐associated changes in TF networks of naïve CD4 T cells appeared to contribute to this differentiation bias. Upon activation, older naïve CD4 T cells expressed increased cell surface density of the transforming growth factor β receptor 3 (TGFβR3) resulting in higher PU.1 expression upon TGFβ stimulation. Moreover, expression of BATF and IRF4 was increased and expression of BCL6 and ID3 was reduced. The concerted activity of these changes favored the differentiation of older naïve CD4 T cells into TH9 cells.

## 2 RESULTS

### 2.1 Aged naïve CD4 T cells are biased toward TH9 differentiation

**Figure 1:**
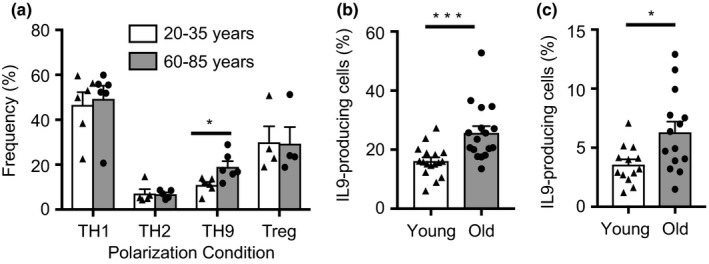
Aging‐associated increase in TH9 lineage commitment. (a) Purified naïve CD4 T cells from 20–35 (young) and 60–85 (old) year‐old adults were cultured under TH1, TH2, TH9, and Treg polarization condition. Frequencies of lineage‐committed T cells were determined by flow cytometry for IFNγ (TH1), IL4 (TH2), and IL9 (TH9) after PMA/ionomycin restimulation on day 7, or for FOXP3 (Treg). Results are shown as mean ± *SEM* from 4–6 experiments. (b) Confirmatory results of TH9 cell polarization experiments with naïve CD4 cells from 17 young and 17 old donors. (c) Frequencies of IL9‐producing cells in total CD4 T cells from 13 young and 14 old donors on day 3 after activation. All results are shown as dot plots with bars indicating mean ± *SEM*. Comparisons were done by two‐tailed *t* test; *p < 0.05; ***p < 0.001

To determine whether T‐cell aging induces epigenetic and transcriptional changes that influence lineage differentiation, we purified naïve CD4 T cells from peripheral blood mononuclear cells and cultured them under lineage‐specific polarization conditions for 7 days. While we did not detect age‐associated differences in TH1, TH2, or Treg differentiation, the frequency of IL9‐producing cells was increased by about 50% (Figure 1a). We confirmed the initial screening experiments by comparing TH9 polarization of naïve CD4 T cells from <35 and >60‐year‐old adults in 17 independent experiments (Figure 1b, *p* < 0.001). About half of these samples were obtained from blood donations, the other half from healthy volunteers, with results being similar. A predisposition of naïve CD4 T cells to develop into TH9 cells should eventually result in the enrichment of TH9 T cells in the memory cell pool. Thus, we compared IL9 production by purified CD4 T cells from 13 young and 14 older adults on day 3 after stimulation, a time point when IL9 derives from memory and is not detectable for naïve CD4 T cells. The mean frequency of IL9‐producing cells was approximately two‐fold higher in the older individuals (Figure 1c).

### 2.2 Age‐associated increase in TGFβ responsiveness favors TH9 lineage commitment

**Figure 2:**
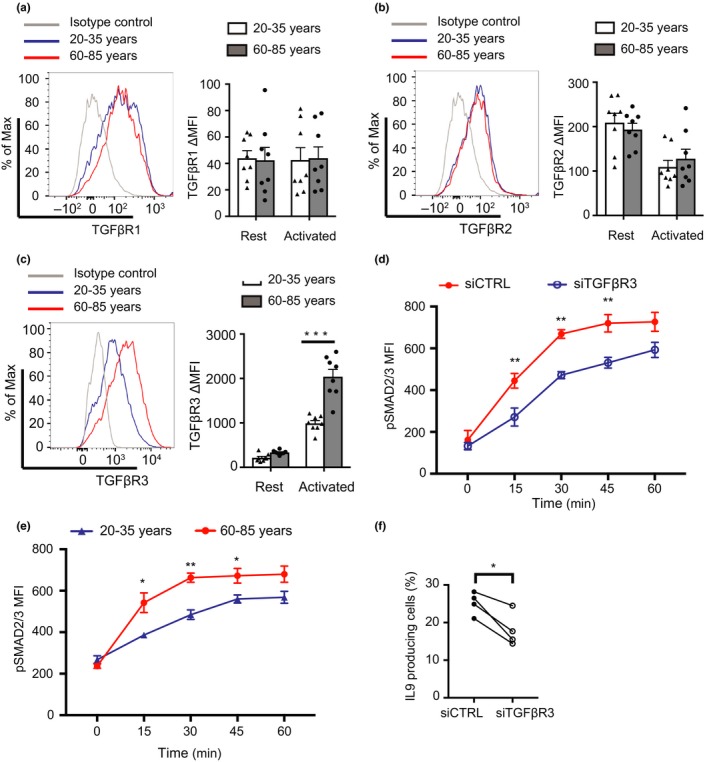
Increased expression of TGFβR3 on activated naïve CD4 T cells with age. Expression of TGFβR1 (a), TGFβR2 (b), and TGFβR3 (c) on naïve CD4 T cells from young and older donors. Cells were either nonstimulated or anti‐CD3/anti‐CD28 beads‐activated for 3 days. Results are shown as representative histograms or mean ± *SEM* of ΔMFI (subtracting the MFI of the FMO control from the experimental MFI, see Table S1) from eight experiments. Comparisons were done by two‐tailed *t* test, ***p < 0.001. (d) Purified naïve CD4 T cells from old donors were activated with anti‐CD3/anti‐CD28 beads for 3 days, then transfected with siRNA for TGFβR3 or control siRNA. SMAD2/3 phosphorylation after TGFβ1 stimulation was determined on day 5. Histograms showing efficiency of TGFβR3 knockdown are shown in Figure S3. (e) Time course of SMAD2/3 phosphorylation after TGFβ1 stimulation. Naïve CD4 T cells from four young and four old donors were activated with anti‐CD3/anti‐CD28 beads at a 1:5 ratio, TGFβ1 stimulation was done on day 3. Results in (d) and (e) are shown as mean ± *SEM* and were compared by paired (d) and unpaired (e) two‐tailed *t* test. (f) Purified naïve CD4 T cells from four old adults were cultured under TH9 polarization condition and transfected with siRNA for TGFβR3 or control siRNA. Cells were restimulated and analyzed by intracellular staining for IL9. Frequencies are compared by paired *t* test. *p < 0.05. **p < 0.01

TH9 cells develop from naïve T cells in the presence of TGFβ and IL4, dependent on a TF network including PU.1, which is downstream of TGFβ, and STAT6, IRF4, and GATA3, which are downstream of IL4 (Kaplan, 2016). TGFβ activity is required for TH9 polarization at least up to 5 days of the culture period (Figure S1). We, therefore, determined the expression of TGFβ receptors on naïve CD4 T cells before and 3 days after activation. At neither time point did we observe an age‐associated difference in the expression of TGFβR1 or TGFβR2 (Figure 2a,b). In contrast, the upregulation of TGFβR3 was about two‐fold enhanced in naïve CD4 T cells from old individuals (Figure 2c). Kinetically, TGFβR3 was highly induced by T‐cell activation peaking on day 3 and then declining again (Figure S2). Silencing of TGFβR3 showed the functional importance of its cell surface density for SMAD2/3 phosphorylation (Figure 2d). TGFβR3‐silenced activated naïve CD4 T cells from older individuals had a slower and reduced response to TGFβ than control siRNA‐treated T cells. Consistent with the role of TGFβR3 in SMAD signaling, activated naïve CD4 T cells from older individuals also had increased SMAD2/3 phosphorylation (Figure 2e). Moreover, reducing expression of TGFβR3 by half in naïve CD4 T cells from older individuals decreased TH9 polarization (Figure 2f).

**Figure 3:**
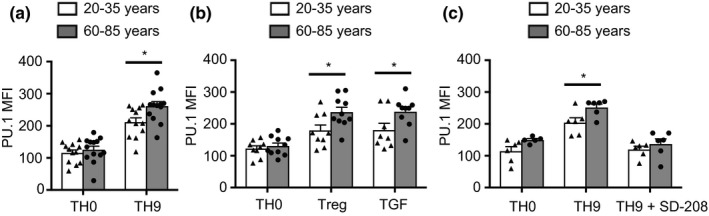
Increased TGFβ‐induced PU.1 expression with age. (a–c) PU.1 expression was determined by flow cytometry of naïve CD4 T cells activated by anti‐CD3/anti‐CD28 beads (1:5) and cultured under indicated condition for 5 days. Results from 12 young and 13 old donors are shown for TH9 polarization (a), nine young and nine old donors cultured with IL2 and TGFβ (Treg) or TGFβ solely (b), and six experiments under TH9 condition with and without the TGFβ receptor inhibitor SD‐208 at a concentration of 1 μM (c). TH0 were activated with anti‐CD3/anti‐CD28 beads without any additional cytokines. All results are shown as mean ± *SEM*. Comparisons were done by two‐tailed *t* test, *p < 0.05

TGFβ functions by inducing the expression of the TF PU.1 required for TH9 differentiation (Chang et al., 2010; Veldhoen et al., 2008). In longitudinal studies, PU.1 expression was first observed between day 3 and 4 of polarization (Figure S4). Thus, we compared PU.1 on day 5 by flow cytometry in activated naïve CD4 T cells. We found about 30% increased expression in aged T cells (Figure 3a). This increased expression was related to TGFβ responsiveness, because it was also seen under Treg polarizing conditions and with TGFβ alone (Figure 3b). Moreover, PU.1 expression was sensitive to treatment with the TGFβR inhibitor SD‐208 (Figure 3c).

### 2.3 Age‐associated bias in TF networks favors TH9 lineage commitment

**Figure 4:**
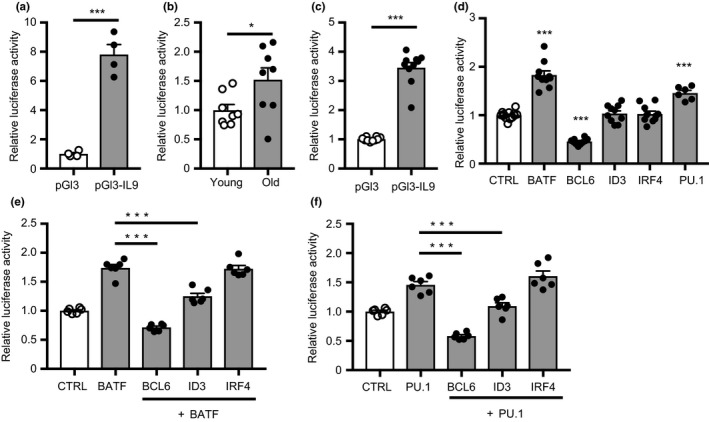
Increased activity of the IL9 promoter with age. (a and b) Purified naïve CD4 T cells were cultured under TH9 polarization condition and co‐transfected with the Renilla luciferase control plasmid and pGL3 plasmid with or without an *IL9* promoter construct. Firefly luciferase activity was normalized to that of Renilla luciferase. Results are shown relative to the mean activity of pGL3 basic plasmid (a, *n* = 4) or relative to the IL9 promoter reporter activity in T cells from young donors (b, *n* = 8). Mean ± *SEM*; comparisons by two‐tailed *t* test. *p < 0.05, ***p < 0.001. (c) Reporter gene activity of the *IL9* promoter in HEK 293T cells relative to the basic plasmid. (d–f) HEK 293T cells were co‐transfected with pGL3‐IL9, Renilla luciferase control plasmids and plasmids expressing the indicated TF. Firefly luciferase activity was measured 48 hr after transfection and normalized to that of Renilla luciferase; the IL9 reporter activity of cells transfected TFs was then normalized to the mean reporter activity of control‐transfected HEK293T. Results shown are means ± *SEM* from 6 to 12 experiments, comparisons were done by one‐way ANOVA and post hoc Tukey. *p < 0.05, ***p < 0.001

The increased TGFβ responsiveness was sufficient to upregulate PU.1 expression, but not to bias T‐cell differentiation toward other TGFβ‐dependent lineages, such as Tregs. We, therefore, explored whether activated aged naïve CD4 T cells preferentially express TFs in addition to PU.1 that are important for IL9 transcription. To identify the gene regulatory regions accounting for the aging‐associated differential IL9 expression, we cloned the human *IL9* promoter region from −406 to +361 bp into the pGL3 plasmid and performed reporter gene assays. Naïve CD4 T cells were cultured under TH9 polarizing conditions and co‐transfected on day 4 with the pGL3‐IL9 promoter and the Renilla luciferase reporter pRL‐TK. Compared to the control pGL3, the sequence of the *IL9* transcription start site (TSS) displayed activity (Figure 4a). In comparing activated naïve CD4 T cells from young and old healthy adults, we found an about 50% increase in reporter activities in older T cells, suggesting that the increased IL9 expression in older T cells was mediated by increased promoter activity (Figure 4b). To identify TFs that may account for this increased activity, we first established that the *IL9* promoter reporter is active in HEK293T cells (Figure 4c). We then overexpressed TFs which have been previously implicated in regulating the *IL9* promoter. Overexpression of BATF increased the activity of the *IL9* promoter reporter by about 2‐fold, overexpression of BCL6 suppressed activity by 50%, while IRF4 and ID3 had no effect (Figure 4d). To examine negative or positive cooperative interactions between the TFs, we co‐transfected BCL6, ID3, or IRF4 with either BATF (Figure 4e) or PU.1 (Figure 4f). In both the settings, BCL6 and ID3 suppressed reporter gene activity while IRF4 again had no effect.

**Figure 5:**
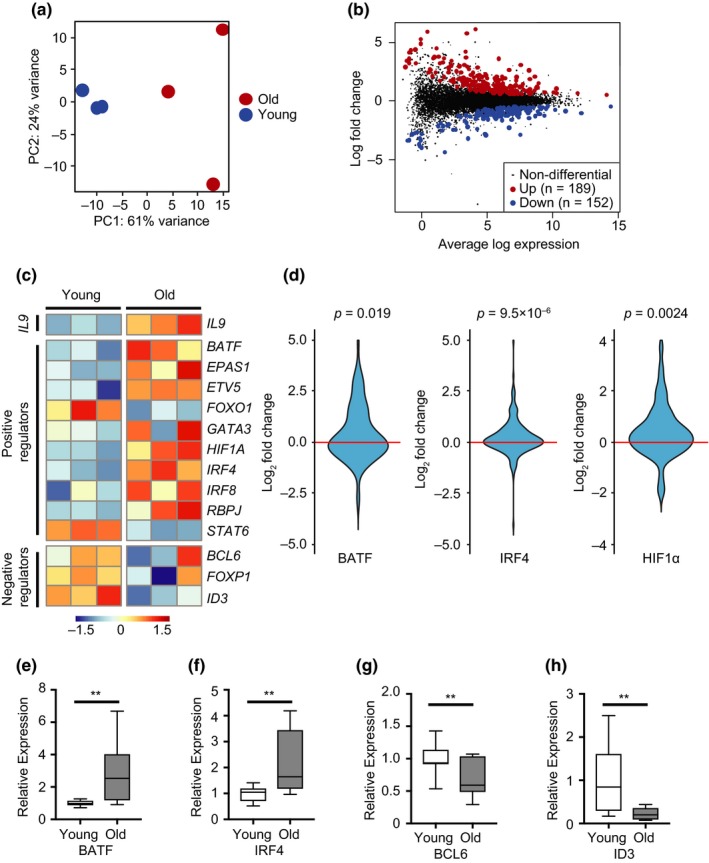
Transcription factor networks regulating IL9 expression in activated old naïve CD4 T cells. Naïve CD4 T cells were stimulated with anti‐CD3/anti‐CD28 beads and cultured for 5 days under nonpolarizing conditions. Transcriptomes of activated cells from three young and three adults older than 60 years were generated by RNA‐seq. (a) Principal Component Analysis of the top 500 most variable genes between young and old activated CD4 T cells. (b) MA plot comparing transcriptomes of CD4 T cells from young and older adults. Colored dots indicate differential expression with Benjamini–Hochberg adjusted *p*‐values of <0.01. Differentially expressed genes are listed in Table S2. (c) Heat map showing the relative gene expression of IL9 and IL9 transcriptional regulators. (d) Violin plots showing the fold gene expression differences of BATF, IRF4 and HIF1α target genes, comparing the transcriptome of old to young T cells. Indicated target sets are from published ChIP‐seq, expression data or experimental data (Benita et al., 2009; Kurachi et al., 2014; Shaffer et al., 2008). Statistical analysis by Wilcoxon rank sum tests. Genes with the largest aging‐related increase are given in Table S3. (e–h) Gene expression of indicated TF by activated naïve CD4 T cells cultured under TH9 polarizing conditions were determined by qPCR. Results are shown normalized to *GAPDH* expression and relative to the mean expression in young adults. Box plots show data from 11 experiments; statistical analysis by one‐tailed *t* test. **p < 0.01

To identify differential expression of TFs with age, naïve CD4 T cells from three young and three >60‐year‐old healthy adults were activated by anti‐CD3/anti‐CD28 beads and cultured under nonpolarizing conditions. Transcriptomes were determined by RNA‐seq on day 5 of culture. As shown in the principal component analysis (PCA) of the 500 most variable genes, naïve CD4 T cells from young individuals formed a tight cluster (Figure 5a). Naïve CD4 T cells from older individuals were clearly segregated from those of young adults in PC1 (61% of variance), while being highly heterogeneous in PC2 (24% of variance). About 340 genes were differentially expressed, with an about equal number being higher or lower (Figure 5b, Table S2). Enrichment analysis of differentially expressed genes identified WNT signaling for genes higher expressed in young CD4 T cells (Figure S5a) while genes higher expressed in aged CD4 T cells were enriched for cytokine–cytokine receptor interaction, TNF and NF‐κB pathways (Figure S5b).

The heat map in Figure 5c compares the expression of IL9 and positive and negative regulators previously implicated in *IL9* transcription including those that we found to regulate the *IL9* promoter. Even under these Th0 conditions, at which transcript numbers of PU.1 were too low to be reliably detected, we found increased expression of *IL9* transcripts in older activated naïve T cells. TGFβR3 transcripts were not different, consistent with the finding that TGFβR3 expression on day 5 after activation had already declined (Figure S2). With the exception of *FOXO1* and *STAT6*, all positive IL9‐transcriptional regulators were overexpressed, while the expression of negative regulators was reduced with age. Increased expression of TFs was of functional importance; BATF, IRF4, and HIF1α target genes were all significantly higher in old naïve CD4 T cells (Figure 5d). Age‐associated differential expression of *BATF*, *IRF4*, *BCL6,* and *ID3* transcripts were confirmed by qPCR of activated naïve CD4 T cells from 11 young and 11 older adults (Figure 5e).

**Figure 6:**
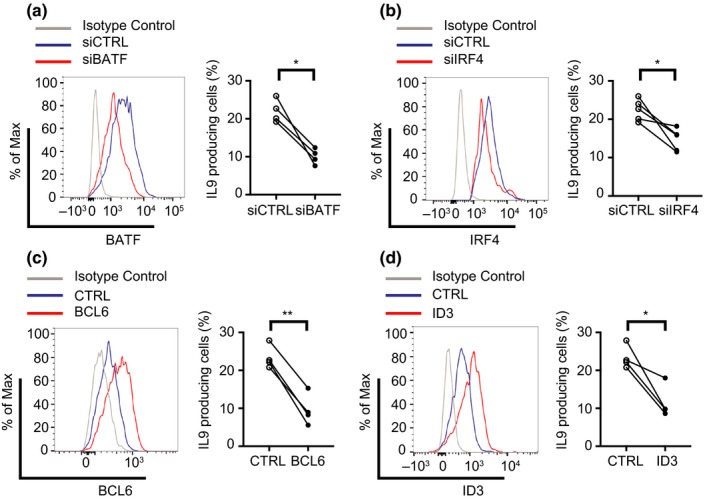
Contribution of the BATF, IRF4, BCL6 and ID3 expression level to the increased aging‐related IL9 expression. Naïve CD4 T cells from old donors were cultured under TH9 polarization condition. Knockdown of BATF (a) and IRF4 (b) after transfection with indicated siRNA. Overexpression of BCL6 (c) and ID3 (d) after transfection with the appropriate plasmids. Transcription factor expression is shown as representative histograms (left). Corresponding frequencies of IL9‐producing CD4 T cells were determined on day 7 after restimulation (right panels). Results from 4 to 5 experiments are shown, statistical analysis by paired *t* test. *p < 0.05, **p < 0.01

To determine whether the age‐associated patterns in TF expression contribute to the TH9 lineage bias observed in older CD4 T cells, we silenced BATF (Figure 6a) and IRF4 (Figure 6b) in naïve CD4 T cells from older individuals cultured under TH9 polarization conditions on day 3 after activation. Silencing reduced TF expression by about 2‐ to 3‐fold by day 5. Reduced expression of either TF significantly reduced the frequency of IL9‐producing cells. Conversely, overexpression of BCL6 and ID3 induced a similar decline in frequencies (Figure 6c,d), identifying these TFs as contributors to the increased TH9 differentiation with age.

## 3 DISCUSSION

In the current study, we examined whether the immune aging process impacts the plasticity of naïve CD4 T cells, thereby influencing their ability to differentiate upon activation. We show that naïve CD4 T cells from older individuals have a higher propensity to develop into TH9 cells. The underlying mechanisms involve changes in cell signaling as well as expression of TF networks that synergize to direct lineage differentiation. Naïve CD4 T cells from older individuals have increased upregulation of TGFβR3 upon activation, endowing the cells with increased responsiveness to TGFβ stimulation. In parallel, older naïve CD4 T cells respond to activation with increased expression of the transcriptional regulators BATF and IRF4, while the repressors BCL6 and ID3 are diminished. These age‐associated changes cooperate to enhance TH9 differentiation.

The mechanisms favoring IL9 production with age is consistent with the current thinking on how TH9 cells are generated and *IL9* transcription is controlled (Kaplan, 2016; Kaplan et al., 2015; Malik & Awasthi, 2018). Of note, a single master regulator for TH9 development has not been identified and lineage specificity appears to be conferred through a combination of TFs, which to varying degree are also involved in the differentiation to other lineages. The observed aging‐associated bias toward TH9 differentiation is, therefore, not explained by a single mechanism.

Naïve T cells are polarized to TH9 cells through culture in the presence of TGFβ and IL4. TGFβ‐induced signaling influences IL9 transcription directly through SMAD2/3 phosphorylation (Tamiya et al., 2013; Wang et al., 2013) and also induces the expression of the TF PU.1 through a noncanonical pathway (Chang et al., 2010; Dardalhon et al., 2008; Kaplan et al., 2015; Veldhoen et al., 2008). TGFβ signaling is mediated by a multi‐molecular receptor complex. Upon TGFβ binding, TGFβR1 and TGFβR2 associate resulting in the phosphorylation of TGFβR1 and of downstream SMAD2/3 (Massague, 2012). TGFβR3 contains a very short cytoplasmic domain without intrinsic enzymatic activity and is therefore not directly involved in the signaling cascade. However, TGFβR3 promotes TGFβR2 binding to and phosphorylation of TGFβR1 in response to TGFβ stimulation (Gatza, Oh, & Blobe, 2010). While expression of TGFβR1 and TGFβR2 was not influenced by age, TGFβR3 was more inducible by T‐cell activation in older individuals. The mechanisms of the increased upregulation of TGFβR3 are undetermined, but aging‐associated changes in microRNA expression may contribute. We have previously examined the effect of age on microRNA profiles of naïve CD8 T cells and have found an enrichment for targets within the TGFβ pathway (Gustafson, Cavanagh, Jin, Weyand, & Goronzy, 2019). In particular, let‐7, a miRNA that has been shown to regulate TGFβR3 expression, decreases with age (Bronevetsky, Burt, & McCune, 2016). Importantly, the increased expression of TGFβR3 was functionally significant, as reducing TGFβR3 dampened SMAD phosphorylation and TH9 differentiation. Moreover, the increased expression of PU.1 in naïve CD4 T cells from older individuals was strictly TGFβ dependent. Our results are consistent with other studies showing that overexpression of TGFβR3 increases the cellular response to TGFβ (Criswell, Dumont, Barnett, & Arteaga, 2008; Nishida, Miyazono, & Ehata, 2018). We, therefore, propose that the increased TGFβR3 expression with age lowers the TGFβ signaling threshold, resulting in increased SMAD phosphorylation as well as increased expression of PU.1.

The increased TGFβ responsiveness with age was not sufficient to increase Treg polarization, emphasizing that this mechanism alone is insufficient to enhance lineage commitment. Although TGFβ‐induced PU.1 has been clearly associated with TH9 differentiation, it is not considered a lineage‐specific master regulator equivalent to T‐bet, GATA3, and RORCγt for TH1, TH2, and TH17 cells, respectively (Kaplan, 2016; Zhu, 2017). Commitment to the TH9 lineage is the result of a number of TFs acting in concert. Recent ChIP‐sequencing studies identified binding of IRF4, BATF, STAT5, STAT6, PU.1, GATA3, and FOXO1 at five different regulatory regions of *IL9* including the promoter (Koh et al., 2018). IL4, required in vitro in addition to TGFβ, promotes TH9 differentiation, in part by pSTAT6 inhibiting TGFβ‐induced FOXP3 and T‐bet expression and therefore inhibiting Treg and TH1 polarization (Dardalhon et al., 2008; Kaplan et al., 2015). However, we did not find evidence for increased STAT6 signaling with age. Moreover, we found reduced STAT6 expression in our transcriptome study of activated T cells. IL4 also functions by inducing IRF4 that binds to the IL9 promoter region together with PU.1 (Chang et al., 2010; Staudt et al., 2010). We found increased expression of IRF4 in activated naïve CD4 T cells from older individuals even when cultured in the absence of exogenous IL4. We did not find an effect of IRF4 in IL9 promoter reporter gene assays in HEK293T, possibly due to the lack of a coactivator that is required for IRF4 binding. In support of the notion that increased IRF4 expression with age is functionally important, reducing the expression of IRF4 expression in naïve CD4 T cells from old individuals significantly reduced TH9 polarization. Of note, IRF4 is not specific to TH9 commitment but also involved in TH2 and TH17 differentiation (Huber & Lohoff, 2014). However, TH2 differentiation was not increased with age, at least not under conditions of optimal TCR stimulation used here, again implying that a combination of aging‐associated differences in TF expression rather than a single TF biases the observed preference in lineage development.

In reporter gene assays, the increased transcription of IL9 in activated and polarized naïve CD4 T cells from older adults was mapped to the IL9 promoter. PU.1 and IRF4 bind to a sequence motif in the promoter, but they were not the only TFs involved in *IL9* transcriptional changes with age. Transcriptome studies of naïve CD4 T cells activated under nonpolarizing conditions showed aging‐associated changes in a multitude of transcriptional activators and repressors that favored TH9 development. Most importantly, we found BATF to be overexpressed, reminiscent to our previous finding in CD8 T cells that increased accessibility to BATF‐binding motifs is an epigenetic hallmark of T‐cell aging (Moskowitz et al., 2017). In murine studies, BATF is important for the majority of TH9‐associated genes, including *IL9*, emphasizing the relevance of this TF (Jabeen et al., 2013). Naïve CD4 T cells from BATF‐deficient mice exhibited impaired TH9 differentiation, while overexpression of BATF enhanced the propensity to produce IL9 (Jabeen et al., 2013). As shown in Figure 6, silencing of BATF in naïve CD4 T cells from older individuals reduced IL9 production when cultured under TH9 polarizing condition.

Other transcriptional activators, which have been described to bind to the IL9 promoter and were found to be overexpressed with age, include HIF1α, GATA3, and ETV5 (Kaplan, 2016; Malik et al., 2017; Singh, Garden, Lang, & Cobb, 2016; Wang et al., 2016). In contrast, BCL6 and ID3, transcriptional repressors of IL9, declined with age. ID3 prevents binding of E2A and GATA3 (Nakatsukasa et al., 2015), while BCL6 competes with STAT5 binding at the promoter region of *IL9* (Bassil et al., 2014). Overexpression of either ID3 or BCL6 in CD4 T cells from older individuals reduced their ability to differentiate into IL9‐producing cells (Figure 6). Again, while important for TH9 differentiation, these TFs in other context are also important for other lineages, for example, BATF is involved in TFH and TH17 differentiation, while BCL6 is required for TFH (Crotty, 2014; Schraml et al., 2009).

Our data on TF networks in naïve CD4 T cells are consistent with the concept that immune aging involves the activation of T‐cell differentiation pathways resulting in a loss of naivety and stemness (Goronzy et al., 2018). Throughout adult life, the T‐cell compartment is maintained by homeostatic proliferation that is under the control of cytokines as well as requires survival signals from TCR (Goronzy & Weyand, 2017; Yanes, Gustafson, Weyand, & Goronzy, 2017). Cytokine‐driven differentiation is a hallmark of virtual memory CD8 cells that accumulate in the mouse with age (Nikolich‐Zugich, 2014). Naïve human T cells exhibit phenotypic changes that are suggestive for partial activation or differentiation (den Braber et al., 2012; Kohler & Thiel, 2009; Pekalski et al., 2017). Also, alterations in microRNA expression patterns with aging are indicative of T‐cell differentiation, including the loss of miR‐181a (Gustafson et al., 2019; Li et al., 2012) and an increase in miR‐21 that favors the activation of TF networks characterized by the reduction in TCF7, BCL6, and ID3 (Kim et al., 2018). Finally, our studies on the epigenome of human naïve CD8 T cells have shown an aging‐associated increased accessibility for bZIP family TFs, including BATF, which is reminiscent of effector cells (Moskowitz et al., 2017).

Previous studies in stem cells have provided evidence for the concept that the aging process involves the activation of TF networks, which limit or influence the differentiation potential (de Haan & Lazare, 2018). The primary example is the switch from lymphoid to myeloid lineage propensity in stem cells with age, again related to the expression of BATF (Wang et al., 2012). In this model, aging is the consequence of the activation of physiological differentiation pathways. Our studies extend this model to T‐cell aging. A functional consequence is a loss in plasticity that is inherent to naïve T‐cell function.

The implications of an increased TH9 propensity for aging are less clear. So far, the relative importance of TH9 cells in vivo has been difficult to define. TH9 cells require antigen‐driven activation to produce IL9, which may explain why IL9 serum concentrations are not increased with age (Whiting et al., 2015). Tissue distribution and activation of TH9 cells will determine their impact. So far, TH9 cells are preferentially found in the skin and are recruited to mucosal surfaces (Clark & Schlapbach, 2017; Stanko et al., 2018). IL9 receptor‐expressing cells include T cells, mainly of the CD4 subtype, hematopoietic precursor cells, mast cells, epithelial cells, and airway smooth muscle cells (Kaplan et al., 2015). As a growth factor, IL9 supports erythroid hematopoiesis as well as T‐cell homeostasis, but also mast cell proliferation (Kaplan et al., 2015). Indeed, mast cells in the skin have been shown to increase with age and contribute to skin aging (Gunin, Kornilova, Vasilieva, & Petrov, 2011). In the intestine, IL9 has a negative impact on epithelial proliferation and repair mechanisms increasing intestinal permeability (Vyas & Goswami, 2018; Weigmann & Neurath, 2017). IL9 has, therefore, been proposed to contribute to the disease manifestations of inflammatory bowel syndrome (Vyas & Goswami, 2018). Along the same lines, it could be a causal factor for decreased mucosal barrier function with age. Thus, depending on the context and the cell type, IL9‐producing cells can have beneficial as well as detrimental effects in older individuals.

## 4 EXPERIMENTAL PROCEDURES

### 4.1 Study population and cells

Peripheral blood and leukapheresis samples were obtained from 85 blood donors in the age ranges of 20–35 and 60–85 years. All of these individuals were healthy, meeting the Stanford Blood bank criteria for blood donation. Samples had been de‐identified except for age range. For the experiments in Figure 1, we collected blood from additional seven female and six male young and old volunteers, who did not have an acute or a poorly controlled chronic disease and who were not frail. The study was approved by the Stanford Institutional Review Board, and all participants gave informed consent. CD4 T cells were isolated with Human CD4 T Cell Enrichment Cocktail (STEMCELL Technologies). Naïve CD4 T cells were further purified by negative selection with CD45RO microbeads by magnetic‐activated cell sorting (Miltenyi Biotec). Purity of CD3^+^CD4^+^CD45RA^+^CCR7^+^ cells was confirmed to be higher than 95% by flow analysis (Figure S6).

### 4.2 In vitro T‐cell differentiation

Isolated naïve CD4 T cells were activated with anti‐CD3/anti‐CD28 beads (Life Technologies) at a 1:5 ratio and cultured for 7 days under the following conditions: TH1, 10 ng/ml IL12 and 1 μg/ml neutralizing anti‐IL4 antibody (Ab); TH2, 20 ng/ml IL4 and 10 μg/ml neutralizing anti‐IFNγ Ab; and TH9, 10 ng/ml IL4 and 5 ng/ml TGFβ1. To generate Tregs, naïve CD4 T cells were cultured on plates coated with anti‐CD3 (5 μg/ml) and anti‐CD28 antibody (1 μg/ml) in the presence of IL2 (2 ng/ml) and TGFβ1 (5 ng/ml). All cytokines were purchased from PeproTech. All antibodies were from R&D Systems. TGFβ receptor inhibitor SD‐208 was purchased from Selleck Chemicals.

siRNA for TGFβR3, BATF, IRF4, and plasmids expressing BCL6 or ID3 were transfected into naïve CD4 T cells on day 3 after activation using the Amaxa Nucleofector system and P3 primary cell Nucleofector Kit (Lonza). TF expressions were examined on day 5 and the frequencies of cytokine‐producing cells were determined on day 7 after restimulation. siRNA were purchased from Dharmacon Inc. and plasmids were from Genscript.

### 4.3 Flow cytometry

Cells were stimulated with 50 ng/ml phorbol 12‐myristate 13‐acetate (PMA; Sigma), 750 ng/ml ionomycin (Sigma) for 4 hr in the presence of GolgiPlug (BD Biosciences). Cells were then stained with anti‐CD4 Ab and LIVE/DEAD™ Fixable Violet Dead Cell Stain Kit, followed by standard fixation and permeabilization according to the manufacturer's protocol (BD Biosciences) and stained with antibodies to IL9, IFNγ, and IL4 (BD Biosciences). Phosflow was performed using Cytofix Buffer and Perm Buffer III following the BD Phosflow™ protocol (BD Biosciences). Cells were treated with TGFβ1 (5 ng/ml) for indicated times, fixed with warm Cytofix Buffer containing phosphatase inhibitor, followed by permeabilization and staining with Ab to pSMAD2/3 (BD Biosciences). Data were collected with a BD LSR Fortessa and analyzed by flowjo software.

### 4.4 qPCR

Total RNA was isolated using RNeasy Plus Micro Kit (Qiagen). cDNA was synthesized with reverse transcriptase (Promega). Transcripts of target genes were quantified by SYBR quantitative PCR on the ABI 7900HT system (Applied Biosystems). Primers used for qPCR were as follows: *BATF*, F‐GACAGAGGCAGACACAGAAG, R‐ GACGGAAATGCAGGTTACACT; *BCL6*, F‐AGAAGCCCTATCCCTGTGAA, R‐ GACGGAAATGCAGGTTACACT; *ID3*, F‐TCATCTCCAACGACAAAAGG, R‐ ACCAGGTTTAGTCTCCAGGAA; *IRF4*, F‐GAGAACGAGGAGAAGAGCATC, R‐CTTTCCTTTAAACAGTGCCCAAG; *GAPDH*, F‐CTCTCTGCTCCTCCTGTTC, R‐ GCGCCCAATACGACCAA. The *C*_t_ values were normalized to *GAPDH*. Results are shown as relative expression compared to the average expression in young adults.

### 4.5 RNA‐seq and downstream analysis

Naïve CD4 T cells were activated with anti‐CD3/anti‐CD28 beads at a 1:5 ratio and cultured for 5 days. Total RNA was isolated using RNeasy Plus Micro Kit (Qiagen), RNA quality was checked with a 2100 Bioanalyzer (Agilent Technologies). Libraries were prepared with Ovation Human FFPE RNA‐Seq Library Systems (NuGEN), quantified using a KAPA Library Quantification Kit (Kapa Biosystems), and sequenced on an Illumina NextSeq 500 at the Stanford Functional Genomics Facility. RNA‐seq reads were aligned to hg19 with STAR. Principal component analysis was run based on the top 500 most variable genes. Differential expression was tested using Limma/edgeR after removing genes with low number reads. Genes were considered to be differentially expressed if Benjamini–Hochberg adjusted *p*‐values were <0.01. Pathway enrichment analysis was performed using DAVID with genes significantly different. EASE Score (a modified Fisher Exact *p*‐value) was used to assess the quality of the enrichment with score <0.1 considered as enriched. Sequence data are available from the SRA database under accession number SRP158502. TF target genes were obtained from published ChIP‐seq (BATF), expression data (IRF4), or experimental data (HIF1α; Benita et al., 2009; Kurachi et al., 2014; Shaffer et al., 2008).

### 4.6 Reporter gene assays

The human IL9 promoter region from −406 to +361 bp was cloned into a pGL3‐basic plasmid. pGL3 basic plasmids or pGL3‐IL9, the Renilla luciferase reporter pR‐TK, and the indicated genes were co‐transfected into HEK 293T cells using FuGENE HD Transfection Reagent (Promega). Forty‐eight hours after transfection, luciferase activity was determined using the dual luciferase reporter assay system (Promega). Alternatively, pGL3‐IL9 and the Renilla luciferase reporter pR‐TK were transfected into naïve CD4 T cells on day 4 after activation using the Amaxa Nucleofector system and P3 primary cell Nucleofector Kit (Lonza). Three days after transfection, luciferase activity was determined.

### 4.7 Statistical analysis

Statistical analysis was performed using graphpad prism 7.0 employing paired and unpaired two‐tailed *t* test, Wilcoxon rank sum tests, or one‐way ANOVA, when appropriate; a *p*‐value of <0.05 was considered significant. Sample sizes between 10 and 20 samples per group for two‐group comparisons were chosen. A total of 20 samples per group provide 80% power for detecting a between‐group difference of 0.9 *SD* and 10 samples per group for detecting 1.3 *SD* at the significance level of 0.05 based on the two‐sample *t* test. All data are presented as mean ± *SEM*.

## CONFLICT OF INTEREST

None declared.

## AUTHOR CONTRIBUTIONS

BH, GL, CEG, CMW, and JJG designed research and analyzed data. BH, GL, and ZY performed the experimental work. TL performed the statistical analysis. BH, CMV, and JJG wrote the manuscript.
