# Supplementary material for: Transcription factor networks in aged naïve CD4 T cells bias lineage differentiation
Source: Aging Cell. 2019 Jul 1;18(4):e12957. doi: 10.1111/acel.12957 (PMC6612640; doi:10.1111/acel.12957)
Supplement: Supplementary file 1 [file ACEL-18-e12957-s001.docx]

**Supporting Information**

**Figure S1. Requirement for TGFβ activity throughout TH9 polarization.** Purified naïve CD4 T cells were cultured under TH9 polarization condition with (gray bars) or without (white bars) the TGFβ receptor inhibitor SD-208; the inhibitor was added at indicated days. Cells were restimulated with PMA/ionomycin and analyzed by intracellular staining for IL9 on day 7. Results are shown as mean ± SEM from 4 experiments and were compared by one-way ANOVA and post-hoc Tukey. **p < 0.01, ***p < 0.001.

**Figure S2. Kinetics of TGFβR3 expression after T cell activation.** Purified naïve CD4 T cells were activated by anti-CD3/anti-CD28 beads (1:5); expression of TGFβR3 was determined at indicated days by flow cytometry. Results shown as mean ± SEM of ΔMFI (subtracting FMO from MFI) are from four donors.

**Figure S3. Knockdown efficiency of *TGFBR3* siRNA.** Purified naïve CD4 T cells from older donors were activated with anti-CD3/anti-CD28 beads for 3 days, then transfected with siRNA for *TGFBR3* or control siRNA. TGFβR3 expression level was determined on day 5. Partial silencing was equivalent to the difference between young and old T cells.

**Figure S4. Kinetics of PU.1 expression under TH9 polarization.**  Purified naïve CD4 T cells were activated by anti-CD3/anti-CD28 beads (1:5) and cultured under TH9 condition, cells were fixed at indicated days and PU.1 expression was determined by flow cytometry. Results are shown as mean ± SEM from two donors.

**Figure S5. Pathway enrichment analysis on differentially expressed genes**

Enrichment analysis of differentially expressed genes (Table S2) was performed using the DAVID Functional Annotation Tool and the KEGG pathway database. A modified Fisher Exact P-Value was used to assess the enrichment, the top 5 enriched pathways for genes more expressed in young (A) and old (B) are shown.

**Figure S6.** **Purity of isolated naïve CD4 T cells**

Naïve (CD3^+^CD4^+^CD45RA^+^CCR7^+^) CD4 cells were isolated from blood of health donors by negatively selection with Human CD4 T Cell Enrichment Cocktail kit and CD45RO magnetic microbeads. The purity of naïve CD4 cells were confirmed by flow cytometry.

**Table S1. Flow cytometric analysis of TGFβR3 expression**

Expression of TGFβR3 on naïve CD4 T cells from young and old donors unstimulated or stimulated for three days. Raw data for MFI (mean fluorescence intensity) and FMO (fluorescence minus one) to calculate ΔMFI shown in Figure 2C.

| **Donors** | **Unstimulated** | | | **Activated** | | |
| --- | --- | --- | --- | --- | --- | --- |
|  | **MFI** | **FMO** | **ΔMFI** | **MFI** | **FMO** | **ΔMFI** |
| Young_1 | 562 | 175 | 387 | 1233 | 252 | 981 |
| Young_2 | 426 | 135 | 291 | 891 | 238 | 653 |
| Young_3 | 364 | 161 | 203 | 1452 | 298 | 1154 |
| Young_4 | 327 | 205 | 122 | 1324 | 317 | 1007 |
| Young_5 | 366 | 166 | 200 | 1218 | 321 | 897 |
| Young_6 | 324 | 165 | 159 | 1456 | 343 | 1113 |
| Young_7 | 341 | 190 | 151 | 1494 | 280 | 1214 |
| Young_8 | 344 | 139 | 205 | 1258 | 363 | 895 |
| Old_1 | 375 | 116 | 259 | 1472 | 233 | 1239 |
| Old_2 | 463 | 127 | 336 | 1962 | 253 | 1709 |
| Old_3 | 573 | 153 | 420 | 2876 | 278 | 2598 |
| Old_4 | 524 | 159 | 365 | 2794 | 317 | 2477 |
| Old_5 | 482 | 163 | 319 | 2241 | 294 | 1947 |
| Old_6 | 486 | 165 | 321 | 2671 | 316 | 2355 |
| Old_7 | 518 | 207 | 311 | 2688 | 421 | 2267 |
| Old_8 | 495 | 149 | 346 | 2009 | 284 | 1725 |

**Table S2. Genes differentially expressed in young and aged activated CD4 T cells**

Naïve CD4 T cells were stimulated with anti-CD3/anti-CD28 beads and cultured for five days under non-polarizing conditions. Transcriptomes of activated cells from three young and three adults older than 60 years were generated by RNA-seq (see Figure 5). Differentially expressed genes with Benjamini-Hochberg adjusted p-values of less than 0.01 are listed alphabetically.

**Genes with increased expression in young activated CD4 T cells**

| **Gene** | **log_2_(fold-difference)** | **adj. p-value** |
| --- | --- | --- |
| ABLIM1 | -1.32 | 6.46E-03 |
| ACE | -4.41 | 6.46E-03 |
| ACSS1 | -1.39 | 9.89E-03 |
| AGO4 | -1.56 | 4.40E-03 |
| AKAP13 | -0.56 | 7.95E-03 |
| AMICA1 | -1.32 | 5.09E-03 |
| AMIGO1 | -2.86 | 6.89E-03 |
| AP1S2 | -0.87 | 7.35E-03 |
| APBB1 | -0.89 | 9.04E-03 |
| APBB1IP | -0.82 | 4.66E-03 |
| ARHGEF1 | -0.56 | 6.79E-03 |
| ARHGEF18 | -0.94 | 8.63E-03 |
| ASPM | -0.50 | 8.70E-03 |
| BACH2 | -1.31 | 8.89E-03 |
| BCAS3 | -1.34 | 6.46E-03 |
| BCL11B | -0.91 | 6.61E-03 |
| BCL9L | -1.04 | 7.37E-03 |
| BRD3 | -1.26 | 3.84E-03 |
| BTBD11 | -1.22 | 9.35E-03 |
| C16orf54 | -1.02 | 4.66E-03 |
| C16orf86 | -3.24 | 9.34E-03 |
| CABLES1 | -2.23 | 6.52E-03 |
| CACNA1C-AS1 | -2.89 | 8.70E-03 |
| CALCOCO1 | -1.30 | 4.35E-03 |
| CAMK4 | -1.67 | 6.46E-03 |
| CBFA2T2 | -0.73 | 9.71E-03 |
| CBLL1 | -0.62 | 7.30E-03 |
| CBX5 | -0.59 | 6.33E-03 |
| CCDC109B | -0.88 | 7.89E-03 |
| CD27 | -0.98 | 6.43E-03 |
| CD59 | -0.91 | 7.13E-03 |
| CD84 | -0.94 | 6.52E-03 |
| CD96 | -1.10 | 3.84E-03 |
| CDCA7 | -0.82 | 6.46E-03 |
| CDCA7L | -0.71 | 6.46E-03 |
| CDR2 | -0.77 | 5.55E-03 |
| CECR1 | -2.96 | 3.51E-03 |
| CHMP1B | -0.72 | 9.04E-03 |
| CLCN3 | -0.63 | 6.43E-03 |
| COG4 | -0.78 | 7.30E-03 |
| CRTAP | -0.96 | 8.89E-03 |
| CSNK2B | -3.73 | 9.36E-03 |
| CTC1 | -0.87 | 6.46E-03 |
| CUX1 | -1.04 | 6.43E-03 |
| DAAM1 | -0.88 | 7.49E-03 |
| DAPK2 | -3.93 | 7.56E-03 |
| DGCR6L | -1.00 | 4.66E-03 |
| DPYSL2 | -0.86 | 9.04E-03 |
| DYRK2 | -1.28 | 2.14E-03 |
| EPB41 | -1.16 | 4.28E-03 |
| EPHX2 | -1.40 | 7.49E-03 |
| ERBB2IP | -0.66 | 4.66E-03 |
| EVI2A | -1.29 | 2.63E-03 |
| FADS1 | -1.09 | 8.63E-03 |
| FAM102A | -1.43 | 3.85E-03 |
| FAM134C | -0.70 | 6.52E-03 |
| FAM21C | -0.85 | 7.38E-03 |
| FAM65B | -0.87 | 9.34E-03 |
| FAM86JP | -2.76 | 9.27E-03 |
| FCGRT | -2.04 | 6.43E-03 |
| FCMR | -2.32 | 1.70E-03 |
| FYB | -1.28 | 5.64E-03 |
| GIMAP6 | -0.96 | 6.08E-03 |
| GNG7 | -4.02 | 7.35E-03 |
| GPR18 | -1.90 | 8.89E-03 |
| GPR52 | -3.38 | 5.64E-03 |
| H2AFV | -0.54 | 7.95E-03 |
| HEMGN | -2.16 | 9.34E-03 |
| HERC2 | -0.61 | 8.89E-03 |
| HIPK2 | -0.82 | 6.46E-03 |
| HIVEP2 | -0.64 | 8.70E-03 |
| HP1BP3 | -0.62 | 7.57E-03 |
| IKZF2 | -2.50 | 7.14E-03 |
| IL17RA | -1.00 | 3.21E-03 |
| ITGA5 | -1.07 | 9.37E-03 |
| JAK1 | -0.74 | 6.46E-03 |
| KIAA0513 | -1.24 | 9.86E-03 |
| KIF15 | -0.63 | 6.46E-03 |
| KLHDC2 | -0.81 | 7.78E-03 |
| LAPTM5 | -0.69 | 4.66E-03 |
| LBH | -1.66 | 2.52E-03 |
| LDLRAD4 | -1.04 | 8.70E-03 |
| LEF1 | -1.17 | 6.47E-03 |
| LNPEP | -0.71 | 8.74E-03 |
| LOC100996286 | -1.40 | 9.78E-03 |
| LOC101929698 | -3.39 | 6.90E-03 |
| LOC728730 | -2.71 | 9.64E-03 |
| LRRC8D | -1.19 | 9.06E-03 |
| MADD | -0.74 | 4.66E-03 |
| MAN2A1 | -0.76 | 9.38E-03 |
| MBNL3 | -1.05 | 3.21E-03 |
| MBP | -0.85 | 3.48E-03 |
| METTL7A | -2.58 | 6.79E-03 |
| MFGE8 | -1.17 | 6.46E-03 |
| MPP7 | -1.52 | 4.06E-03 |
| NAP1L4 | -0.64 | 6.46E-03 |
| NIN | -0.86 | 6.07E-03 |
| NLRC5 | -0.54 | 7.92E-03 |
| NLRP1 | -0.68 | 9.37E-03 |
| NT5E | -1.92 | 9.89E-03 |
| PACS1 | -1.16 | 7.92E-03 |
| PAN2 | -1.22 | 4.06E-03 |
| PCMTD2 | -1.35 | 7.81E-03 |
| PDE3B | -0.86 | 9.36E-03 |
| PDE4D | -0.63 | 7.91E-03 |
| PDP1 | -1.14 | 9.68E-03 |
| PHACTR2 | -0.94 | 3.46E-03 |
| PIK3IP1 | -2.83 | 9.68E-03 |
| PPP2R5C | -1.05 | 9.48E-03 |
| PRKACB | -1.25 | 6.46E-03 |
| QARS | -0.73 | 7.91E-03 |
| RAB37 | -1.62 | 9.86E-03 |
| RASA3 | -1.04 | 5.68E-03 |
| RASSF3 | -1.20 | 2.58E-03 |
| RCAN3 | -0.92 | 7.91E-03 |
| RCSD1 | -0.59 | 7.38E-03 |
| RICTOR | -0.56 | 8.55E-03 |
| RMRP | -1.06 | 1.63E-03 |
| RNA45S5 | -0.49 | 6.43E-03 |
| RNASEH2B | -0.94 | 7.14E-03 |
| RPL13A | -0.83 | 6.46E-03 |
| RPLP0 | -0.47 | 9.89E-03 |
| RTKN2 | -1.10 | 5.30E-03 |
| SARAF | -0.93 | 9.86E-03 |
| SCML4 | -3.24 | 6.46E-03 |
| SEPT6 | -0.69 | 4.66E-03 |
| SEPT9 | -0.78 | 4.00E-03 |
| SH3KBP1 | -0.56 | 7.92E-03 |
| SIGIRR | -1.24 | 6.27E-03 |
| SLAMF6 | -1.24 | 3.46E-03 |
| SMG6 | -0.66 | 9.65E-03 |
| SNRK | -1.14 | 7.57E-03 |
| STK38 | -1.11 | 6.43E-03 |
| SYNE1 | -1.03 | 4.66E-03 |
| TCF7 | -2.91 | 9.37E-03 |
| TECPR1 | -1.13 | 6.07E-03 |
| TMSB4X | -0.90 | 5.55E-03 |
| TP53INP1 | -2.44 | 4.53E-03 |
| TRIO | -1.72 | 5.87E-03 |
| TTC39C | -0.79 | 7.59E-03 |
| TULP4 | -0.66 | 7.38E-03 |
| UBL3 | -1.54 | 9.86E-03 |
| UCP2 | -0.92 | 5.34E-03 |
| VAV3 | -2.14 | 6.46E-03 |
| VSIG1 | -2.49 | 8.55E-03 |
| WDR86-AS1 | -3.38 | 6.46E-03 |
| WIPI2 | -0.61 | 9.38E-03 |
| YPEL2 | -1.58 | 3.21E-03 |
| ZC3H6 | -0.87 | 9.68E-03 |
| ZFP36L2 | -1.48 | 3.46E-03 |
| ZNF154 | -2.18 | 6.08E-03 |
| ZNF671 | -1.29 | 8.90E-03 |

**Genes with increased expression in old activated CD4 T cells**

| **Gene** | **log_2_(fold-difference)** | **adj. p-value** |
| --- | --- | --- |
| ACTB | 0.49 | 6.43E-03 |
| ADAM19 | 2.60 | 3.21E-03 |
| ADD2 | 1.30 | 3.48E-03 |
| AGPAT9 | 2.08 | 6.43E-03 |
| APBA1 | 3.65 | 6.46E-03 |
| APOL6 | 0.54 | 7.30E-03 |
| ARID3B | 1.07 | 9.35E-03 |
| ARID5A | 2.04 | 6.46E-03 |
| ARNTL2 | 0.93 | 6.46E-03 |
| ARPC5L | 0.96 | 4.66E-03 |
| BST2 | 1.74 | 3.21E-03 |
| CCDC58 | 1.24 | 6.89E-03 |
| CCDC59 | 0.81 | 8.70E-03 |
| CCR1 | 3.26 | 4.06E-03 |
| CCR2 | 2.68 | 4.35E-03 |
| CD40LG | 1.89 | 5.33E-03 |
| CD58 | 0.91 | 5.55E-03 |
| CDK6 | 1.18 | 1.70E-03 |
| CEACAM1 | 3.41 | 3.46E-03 |
| CHCHD10 | 1.11 | 7.38E-03 |
| CHORDC1 | 0.89 | 3.84E-03 |
| CHRM4 | 3.45 | 5.39E-03 |
| CHSY1 | 1.01 | 7.30E-03 |
| CISH | 3.56 | 4.40E-03 |
| CMAHP | 0.93 | 3.46E-03 |
| COX17 | 0.77 | 7.92E-03 |
| CRYBG3 | 0.98 | 4.18E-03 |
| CSF1 | 2.36 | 1.70E-03 |
| CSF2 | 5.88 | 1.63E-03 |
| CSTF2 | 0.93 | 4.66E-03 |
| CTSC | 0.84 | 6.68E-03 |
| CTSW | 1.52 | 9.60E-03 |
| CXCL8 | 4.34 | 4.40E-03 |
| CXCR4 | 1.18 | 6.46E-03 |
| CYTIP | 1.09 | 7.38E-03 |
| DENND3 | 1.60 | 7.52E-03 |
| DESI1 | 1.09 | 3.99E-03 |
| DGKG | 2.66 | 3.84E-03 |
| DNAJC6 | 1.92 | 3.21E-03 |
| DOK5 | 5.24 | 3.46E-03 |
| DUSP6 | 2.81 | 4.27E-03 |
| EBNA1BP2 | 0.70 | 9.19E-03 |
| EGR1 | 1.72 | 6.46E-03 |
| EHD4 | 1.39 | 3.21E-03 |
| EIF2AK2 | 1.31 | 6.46E-03 |
| EIF4G1 | 0.70 | 3.48E-03 |
| ELL2 | 1.17 | 6.35E-03 |
| EMILIN2 | 2.00 | 4.28E-03 |
| EMP1 | 1.81 | 2.52E-03 |
| ENO1 | 0.63 | 8.69E-03 |
| EPHB1 | 3.16 | 9.38E-03 |
| ESF1 | 0.89 | 7.14E-03 |
| FAM129B | 1.97 | 5.14E-03 |
| FAM3C | 0.91 | 8.69E-03 |
| FGFRL1 | 1.75 | 3.64E-03 |
| FN1 | 3.71 | 1.70E-03 |
| FOSL1 | 3.62 | 1.80E-03 |
| FRMD4B | 1.92 | 4.66E-03 |
| FURIN | 2.03 | 1.63E-03 |
| GADD45GIP1 | 0.75 | 9.34E-03 |
| GNAL | 2.85 | 9.04E-03 |
| GNGT2 | 1.15 | 8.63E-03 |
| GPT2 | 1.59 | 6.82E-03 |
| GREM2 | 3.60 | 9.89E-03 |
| GZMB | 3.04 | 3.46E-03 |
| HAVCR2 | 1.39 | 3.46E-03 |
| HELZ2 | 4.16 | 4.66E-03 |
| HEMK1 | 1.39 | 6.46E-03 |
| HK2 | 1.73 | 1.70E-03 |
| HSP90AB1 | 1.02 | 6.43E-03 |
| ICAM1 | 2.18 | 3.79E-03 |
| IKZF4 | 1.15 | 4.66E-03 |
| IL18RAP | 1.57 | 6.52E-03 |
| IL2RA | 2.57 | 2.58E-03 |
| IL9 | 4.27 | 7.91E-03 |
| IRF4 | 1.46 | 4.28E-03 |
| JAKMIP1 | 1.30 | 8.55E-03 |
| JAM2 | 3.16 | 9.71E-03 |
| JUNB | 1.39 | 4.49E-03 |
| KIAA0020 | 0.77 | 8.79E-03 |
| KIAA1217 | 2.39 | 4.06E-03 |
| LAP3 | 1.24 | 6.43E-03 |
| LDOC1 | 2.41 | 4.06E-03 |
| LIF | 6.09 | 3.21E-03 |
| LINC00152 | 0.78 | 7.38E-03 |
| LINC00892 | 3.72 | 3.48E-03 |
| LINC01132 | 1.74 | 6.46E-03 |
| LRFN4 | 1.68 | 4.06E-03 |
| LSM6 | 0.66 | 9.18E-03 |
| LTA | 2.39 | 4.35E-03 |
| LUC7L3 | 0.59 | 7.30E-03 |
| MAF | 2.06 | 8.69E-03 |
| MAN2A2 | 0.91 | 7.92E-03 |
| MAP1A | 1.61 | 3.46E-03 |
| MAPKAPK3 | 1.13 | 8.63E-03 |
| MB21D1 | 1.28 | 8.75E-03 |
| MB21D2 | 2.81 | 6.89E-03 |
| MINA | 1.44 | 3.30E-03 |
| MLLT4 | 1.81 | 3.21E-03 |
| MRPL32 | 0.79 | 9.65E-03 |
| MRPL33 | 0.87 | 5.68E-03 |
| MTHFD1L | 1.22 | 4.66E-03 |
| NDFIP2 | 2.16 | 9.37E-03 |
| NDUFAB1 | 0.57 | 7.35E-03 |
| NDUFV2 | 0.69 | 6.46E-03 |
| NFKB2 | 0.78 | 8.80E-03 |
| NFKBIE | 0.92 | 6.46E-03 |
| NOL3 | 2.41 | 9.78E-03 |
| NR4A3 | 2.67 | 5.68E-03 |
| NUPL1 | 0.80 | 6.74E-03 |
| OAS2 | 0.66 | 9.65E-03 |
| OSM | 5.71 | 7.38E-03 |
| PCSK6 | 3.41 | 8.69E-03 |
| PELO | 0.78 | 9.68E-03 |
| PER2 | 1.06 | 9.62E-03 |
| PES1 | 0.81 | 5.55E-03 |
| PHB | 0.56 | 7.92E-03 |
| PHLDA1 | 2.17 | 3.21E-03 |
| PICALM | 0.56 | 6.46E-03 |
| PLP2 | 0.65 | 5.87E-03 |
| POLR3E | 0.65 | 6.52E-03 |
| PPFIBP1 | 1.43 | 9.34E-03 |
| PRDM1 | 1.73 | 7.91E-03 |
| PSMG1 | 0.66 | 8.55E-03 |
| PTGFRN | 1.44 | 8.74E-03 |
| RAB11FIP1 | 1.99 | 7.40E-04 |
| RAD23A | 0.57 | 8.20E-03 |
| RALB | 0.88 | 3.46E-03 |
| RANBP1 | 0.93 | 3.48E-03 |
| RASGRP4 | 1.84 | 7.45E-03 |
| RCAN2 | 1.77 | 4.66E-03 |
| RDH10 | 1.63 | 3.46E-03 |
| RGS16 | 3.16 | 4.66E-03 |
| RNF213 | 0.60 | 5.84E-03 |
| RSAD2 | 3.02 | 1.63E-03 |
| RUNX3 | 0.80 | 7.91E-03 |
| SAMD10 | 0.90 | 8.89E-03 |
| SAMD9L | 1.18 | 3.21E-03 |
| SAMSN1 | 1.27 | 5.10E-03 |
| SDC4 | 2.49 | 3.46E-03 |
| SEC61A2 | 1.27 | 6.46E-03 |
| SEMA4A | 1.67 | 9.78E-03 |
| SLAMF1 | 1.34 | 8.05E-03 |
| SLC1A5 | 1.20 | 9.86E-03 |
| SLC26A4 | 4.45 | 9.38E-03 |
| SLC26A4-AS1 | 4.86 | 1.70E-03 |
| SLC38A5 | 0.88 | 4.00E-03 |
| SLC41A2 | 1.87 | 8.55E-03 |
| SLC9A7 | 1.39 | 7.52E-03 |
| SNRPA1 | 0.69 | 7.45E-03 |
| SOCS1 | 2.26 | 4.66E-03 |
| SOCS3 | 1.83 | 9.86E-03 |
| SOGA3 | 2.93 | 4.06E-03 |
| SPAG1 | 0.76 | 9.37E-03 |
| SPTY2D1 | 0.57 | 9.99E-03 |
| SRM | 1.15 | 6.07E-03 |
| SRPRB | 0.76 | 9.65E-03 |
| SRXN1 | 1.37 | 3.65E-03 |
| STARD4 | 1.30 | 4.66E-03 |
| STIP1 | 0.99 | 4.00E-03 |
| STRIP2 | 1.92 | 7.78E-03 |
| STX3 | 1.82 | 9.45E-03 |
| TAB2 | 0.85 | 3.46E-03 |
| TANK | 0.68 | 8.72E-03 |
| TBC1D24 | 0.94 | 7.45E-03 |
| TCEB1 | 0.69 | 7.22E-03 |
| TFRC | 1.12 | 2.14E-03 |
| THG1L | 0.86 | 5.91E-03 |
| TIAM2 | 2.99 | 3.24E-03 |
| TIPIN | 1.09 | 3.21E-03 |
| TMEM65 | 1.13 | 3.48E-03 |
| TNFRSF8 | 2.01 | 5.10E-03 |
| TNFSF10 | 1.18 | 5.39E-03 |
| TNFSF14 | 1.21 | 7.14E-03 |
| TNS4 | 1.33 | 5.64E-03 |
| TOMM40 | 0.89 | 4.66E-03 |
| TPM4 | 0.68 | 5.91E-03 |
| TRAP1 | 0.56 | 8.90E-03 |
| TSPAN18 | 1.57 | 7.56E-03 |
| TXLNA | 0.66 | 4.66E-03 |
| ULBP1 | 4.08 | 7.30E-03 |
| UNQ6494 | 2.61 | 6.45E-03 |
| UPP1 | 1.63 | 8.63E-03 |
| USP18 | 2.90 | 4.66E-03 |
| WARS | 1.82 | 4.40E-03 |
| WNK3 | 3.57 | 8.90E-03 |
| YARS | 0.75 | 8.55E-03 |
| ZBTB32 | 2.12 | 4.66E-03 |
| ZNF267 | 0.88 | 8.89E-03 |

**Table S3. Increased expression of BATF, IRF4 and HIF1α target genes**

BATF, IRF4 and HIF1α target genes from the violin plots in Figure 5D. Genes are listed that showed a log fold increase of greater than 1.5 in T cells from old individuals.

**BATF target genes**

| **Gene** | **log_2_(fold-increase)** | **adj. p-value** |
| --- | --- | --- |
| IL18RAP | 1.57 | 6.52E-03 |
| IL23R | 1.60 | 7.36E-02 |
| TNFRSF9 | 1.62 | 4.74E-02 |
| PRDM1 | 1.73 | 7.91E-03 |
| HK2 | 1.73 | 1.70E-03 |
| ATF3 | 1.91 | 4.40E-02 |
| CD86 | 2.00 | 2.02E-02 |
| TNFRSF8 | 2.01 | 5.10E-03 |
| LAG3 | 2.06 | 1.45E-02 |
| IL2RA | 2.57 | 2.58E-03 |
| ADAM19 | 2.60 | 3.21E-03 |
| CCR2 | 2.68 | 4.35E-03 |
| IL21 | 2.76 | 1.12E-01 |
| TNFRSF4 | 2.89 | 2.11E-02 |
| GZMB | 3.04 | 3.46E-03 |
| IL1R2 | 3.23 | 4.63E-02 |
| CCR1 | 3.26 | 4.06E-03 |
| CCL3 | 4.95 | 5.16E-02 |

**IRF4 target genes**

| **Gene** | **log_2_(fold-increase)** | **adj. p-value** |
| --- | --- | --- |
| NFIL3 | 1.52 | 7.36E-02 |
| GPT2 | 1.59 | 6.82E-03 |
| PIP5K1B | 1.61 | 4.26E-02 |
| ITPKA | 1.66 | 5.19E-02 |
| BSPRY | 1.66 | 3.44E-02 |
| PRDM1 | 1.73 | 7.91E-03 |
| HK2 | 1.73 | 1.70E-03 |
| PFKFB4 | 1.73 | 4.14E-01 |
| ATF3 | 1.91 | 4.40E-02 |
| TNFRSF8 | 2.01 | 5.10E-03 |
| SETBP1 | 2.16 | 1.23E-01 |
| DUSP5 | 2.35 | 3.20E-02 |
| NAPSA | 2.35 | 7.06E-02 |
| VEGFA | 2.48 | 2.93E-02 |
| GTF2IRD1 | 2.52 | 1.01E-02 |
| TBC1D8B | 2.70 | 5.25E-02 |
| NAPSB | 3.25 | 3.14E-02 |
| CCL3 | 4.95 | 5.16E-02 |

**HIF1α Target genes**

| **Gene** | **log_2_(fold-increase)** | **adj. p-value** |
| --- | --- | --- |
| ADM2 | 1.71 | 1.12E-01 |
| HK2 | 1.73 | 1.70E-03 |
| FURIN | 2.03 | 1.63E-03 |
| DDIT4 | 2.05 | 4.57E-02 |
| VEGFA | 2.48 | 2.93E-02 |
| CDKN1A | 2.93 | 2.98E-02 |
| FN1 | 3.71 | 1.70E-03 |
